# A Case of Euthyroid Graves’ Ophthalmopathy With Negative Thyroid Antibodies

**DOI:** 10.7759/cureus.73527

**Published:** 2024-11-12

**Authors:** Rashid S Abu Helwa, Hiyam H Subeh, Venkata Katreddy, Amir I Ahmed

**Affiliations:** 1 General Practice, Emirates Health Services, Sharjah, ARE; 2 Endocrinology, American Hospital Dubai, Dubai, ARE; 3 Geriatrics, American Hospital Dubai, Dubai, ARE

**Keywords:** euthyroid grave's ophthalmopathy with negative antibodies (egona), euthyroid ophthalmopathy, euthyroid orbitopathy, grave's ophthalmopathy, grave's orbitopathy

## Abstract

Grave's ophthalmopathy is a prevalent complication of Grave's disease. In this case report, we present a case of an 84-year-old female patient with no symptoms of hyperthyroidism except that of Grave's ophthalmopathy. The patient's thyroid profile was normal, and both thyroid receptor antibodies and thyroid-stimulating immunoglobulins were negative. MRI showed thickening of the medial and inferior recti muscles in both eyes. With the absence of masses on MRI, a diagnosis of Grave's ophthalmopathy was highly suspicious. Regular monitoring was done for a year, and the patient remained euthyroid with negative antibodies throughout. The patient was managed symptomatically with the addition of methylprednisolone. No surgical intervention was done.

## Introduction

Graves’ ophthalmopathy (GO), also known as Graves’ orbitopathy, is a well-known cause of sight-threatening eye disease. It is the major extrathyroidal manifestation of Graves’ disease (GD), although it may less frequently occur in patients with chronic autoimmune thyroiditis [1]. Age-adjusted annual incidence in patients with GD is around 25-50% [2]. This chronic inflammatory disease of the orbit affects mostly women during their reproductive ages and causes the presence of proptosis, diplopia, visual disturbances, and in severe cases, visual loss [3,4]. The majority of cases with GO patients would have GD, but some patients would develop it with hypothyroidism or normal thyroid function. Although these different presentations are common and clinicians are aware of their clinical appearance, the presence of thyroid peroxidase (TPOAb) and thyroid-stimulating hormone (TSH) receptor antibodies (TRAbs) allows physicians to diagnose and emphasize the presence of GD [4]. GO, with negative antibodies and normal thyroid function, raises clinical diagnostic and treatment challenges for clinicians [5]. In this case, we report a female patient who presented with GO with normal thyroid functions and without any thyroid antibodies present.

## Case presentation

An 84-year-old female patient, a known case of hypertension and diabetes mellitus type 2, presented to the emergency department with a chief complaint of headache for four days. The headache was mainly located in the right temporal area and was radiating towards the right eye. The pain was rated 10 out of 10 and was associated with nausea and photophobia. Diplopia was present but with no visual limitation. The patient was clinically euthyroid, with no symptoms of hyperthyroidism or hypothyroidism. The patient has no history of trauma. Personal and family history were not significant for any thyroid disease. On examination, the right eye was erythematous with mild proptosis noted, but there was no lid lag or retraction present. The patient had no goiter on inspection and palpation. The patient’s visual acuity was 20/150 in the right eye and 20/50 in the left eye. The tonometry was done, and the intraocular pressure was normal on both sides. A differential diagnosis of either a retrobulbar mass or GO was considered.

Investigations

**Figure 1:**
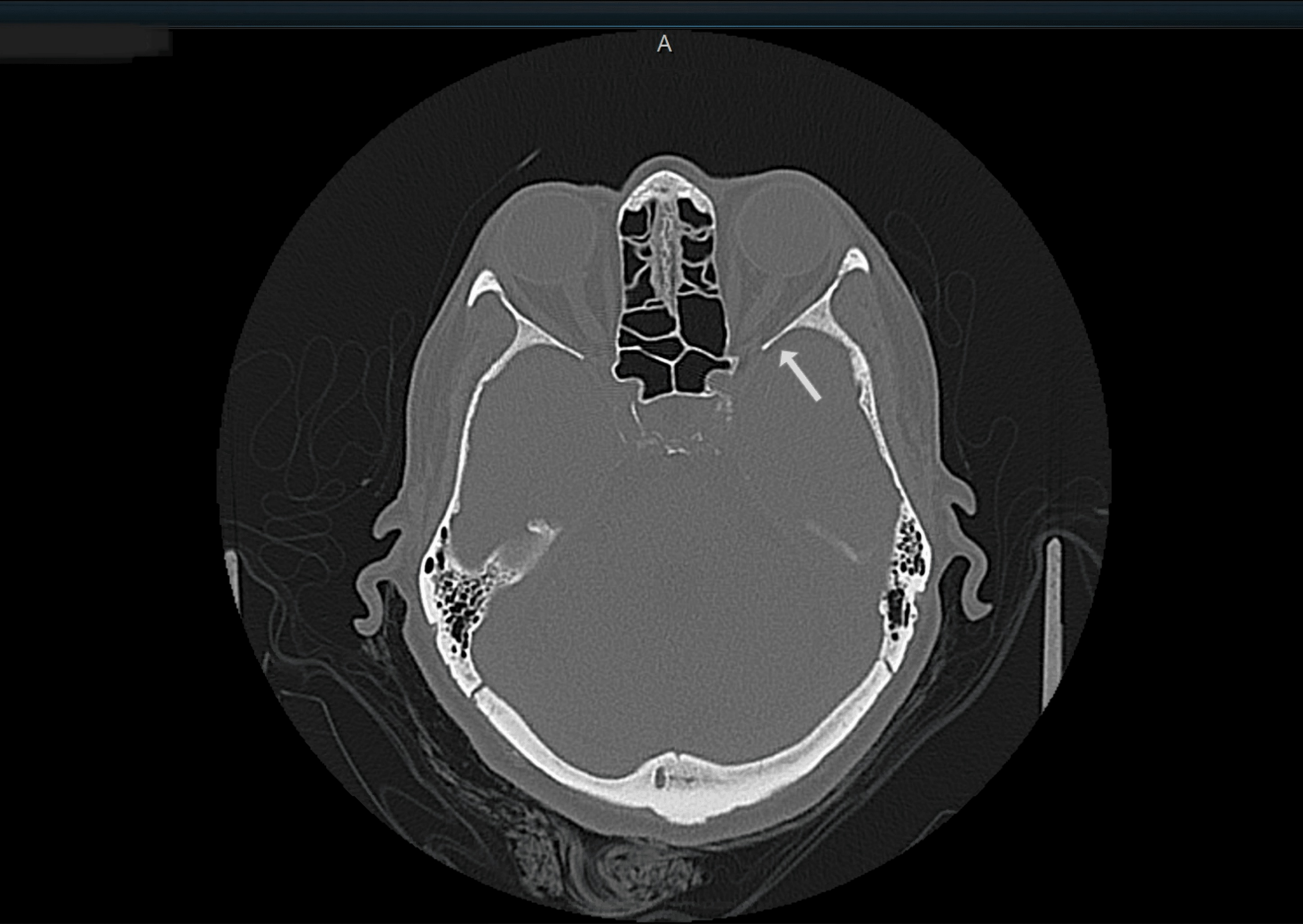
A CT-scan to see any presence of masses in the orbital cavity Checking for any masses present in the left orbital cavity

**Figure 2:**
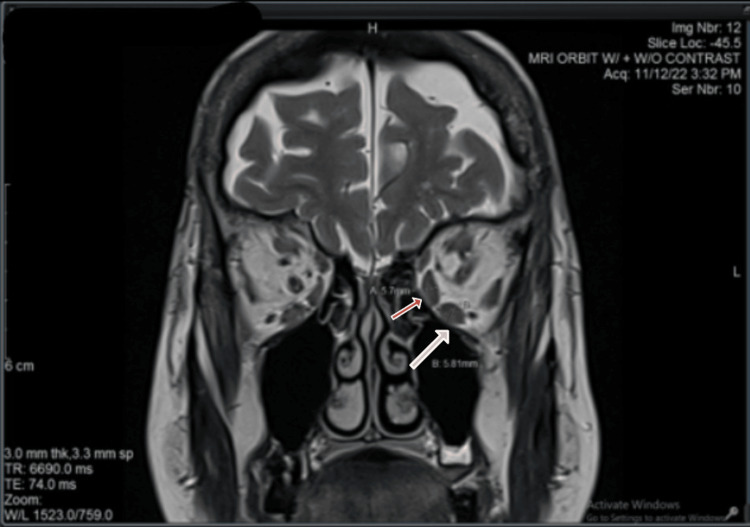
Coronal MRI scan of the extraocular muscles Enlarged left medial and inferior recti muscles measuring at 5.70 mm and 5.81 mm, respectively.

A CT scan of the orbit was performed, which showed no abnormalities (Figure 1). The blood tests demonstrated normal thyroid function; TSH level was 1.25 uIU/ml (reference range: 0.55-4.78) and FT4 was 16.01 pmol/L (reference range: 12-30 pmol/L). The TRAbs and thyroid-stimulating immunoglobulins (TSI) were both negative (Table 1). The MRI scan showed a fusiform thickening on the extraocular muscles, mostly appreciated on the left rather than the right eye. The two most affected muscles by the thickening were the medial and inferior recti. The left medial rectus measured 5.7 mm (normal range: 3.7 ± 0.9 mm), while the left inferior rectus measured 5.8 mm (normal range: 4.0 ± 1.4 mm) (Figure 2). The inflammation and thickening of the rectus muscles in the absence of masses made the diagnosis highly suspicious of GO and ruled out the presence of orbital tumors. Other differential diagnoses such as cavernous sinus thrombosis, orbital cellulitis, and hemorrhage in the orbit were considered unlikely based on the clinical presentation.

**Table 1:** Results indicating normal thyroid function with no evidence of autoimmune thyroid disease, as TRAbs and TSI are negative TRAbs: thyrotropin receptor antibodies; TSI: thyroid-stimulating immunoglobulins

Test	Result	Reference range	Interpretation
Thyroid-stimulating hormone (TSH)	1.25 µIU/mL	0.55-4.78 µIU/mL	Normal
Free thyroxine (FT4)	16.01 pmol/L	12-30 pmol/L	Normal
Thyrotropin receptor antibodies (TRAbs)	Negative	Negative	No thyroid autoantibodies detected
Thyroid-stimulating immunoglobulins (TSI)	Negative	Negative	No thyroid autoantibodies detected

Treatment

The patient’s headache was managed symptomatically using analgesics, mainly opioids, after failure of symptom control with paracetamol and nonsteroidal anti-inflammatory drugs (NSAIDs). The condition was treated medically using methylprednisolone to reduce inflammation of the extraocular muscles. No surgical approach was taken as the thickening was mild and didn’t cause significant diplopia or vision loss. After a week of treatment on high-dose methylprednisolone, the headache was gone completely.

The patient had regular monitoring for about one year. At three months, her decreased visual acuity, diplopia, and proptosis were all gone, and the patient returned to normal. Throughout the one year of follow-up, she remained euthyroid, and her thyroid receptor antibodies and TSI remained negative during follow-up.

## Discussion

Graves ophthalmopathy (GO) is an autoimmune disorder of the orbit that mainly coexists with hyperthyroidism in 80-90% of cases [2]. Euthyroid graves ophthalmopathy (EGO) is a rare form of GO that presents with inflammatory orbitopathy without a present or past history of thyroid dysfunction. It has an estimated prevalence that varies from 0.9% to 15.4% of all who present with grave ophthalmopathy [6]. The clinical features of EGO are usually milder and asymmetrical compared to patients with concurrent hyperthyroidism [7]. The diagnosis of EGO is challenging due to the absence of clinical and biochemical markers of thyroid disease at the time of presentation. As a result, positive serum TRAbs or TPOAbs are used to confirm the diagnosis in such cases [6]. Thus, the clinical presentation of GO without thyroid dysfunction or thyroid-specific autoantibodies presents a diagnostic challenge and warrants consideration of alternative diagnoses [5]. We report a rare case of EGO with negative thyroid autoantibodies to illustrate the importance of radiological assessment in such cases.

The possible factors that can be attributed to EGO with negative thyroid autoantibodies include lower TRAbs titers in euthyroid patients, the specificity of TRAbs and TSI assays, and the complex pathogenesis of GO [5,7]. In a retrospective observational study, the results showed that euthyroid patients have significantly lower TRAbs and TPOAbs titers compared to hyperthyroid patients [7]. According to a cross-sectional descriptive study, the TSI was positive in 84% of patients who were presented with EGO [8]. Furthermore, it’s also important to acknowledge the possible temporal relationship between the onset of ophthalmopathy and thyroid disease. In a few patients, GO precedes the onset of thyroid dysfunction, which necessitates long-term follow-up of those patients. According to a retrospective study, 8-25% of patients develop thyroid abnormalities within 15-45 months of the onset of ophthalmopathy [6].

The most common symptoms that are present in patients complaining of GO are redness and edema of the periorbital tissues as well as upper eyelid retractions [3]. Proptosis is also common in these patients. Our patient did not present with any of those specific findings. Another clinical feature that is present in those patients is pretibial myxedema, which is present in 13% of patients who have a severe form of GO [9], and 20% of those who have pretibial myxedema also present with clubbing of the fingers and toes called thyroid acropathy [3].

The presentation of GO is usually clinically unilateral, unlike what was present with our patient. Orbital imaging showed bilateral changes but with asymmetrical clinical symptoms. On the other hand, our patient presented with a unilateral left ocular muscle enlargement on both muscles of the left medial and left inferior rectus. This caused more confusion as to its unilateral presentation on imaging, which normally is presented bilaterally.

## Conclusions

This case highlighted the importance of GO as a differential diagnosis even in patients with euthyroid and with negative antibodies to delay diagnosis, as patients will receive appropriate treatment to prevent any significant ocular damage and visual loss. More in-depth understanding is needed regarding the manifestation and clinical presentation of GO. Findings and treatment may differ as well, and throughout the literature, no unified way of treating or managing has been finalized due to the lack of cases that are present in the literature. We recommend the unified use of terms to make it easier to compare results and establish means of managing for future upcoming cases.
